# A hidden Markov model for reconstructing animal paths from solar geolocation loggers using templates for light intensity

**DOI:** 10.1186/s40462-015-0062-5

**Published:** 2015-10-15

**Authors:** Eldar Rakhimberdiev, David W. Winkler, Eli Bridge, Nathaniel E. Seavy, Daniel Sheldon, Theunis Piersma, Anatoly Saveliev

**Affiliations:** Department of Ecology and Evolutionary Biology and Laboratory of Ornithology, Cornell University, Ithaca, 14853 USA; Department of Marine Ecology, NIOZ Royal Netherlands Institute for Sea Research, PO Box 59, 1790 AB Den Burg, The Netherlands; Department of Vertebrate Zoology, Biological Faculty, Lomonosov Moscow State University, Moscow, 119991 Russia; Oklahoma Biological Survey, University of Oklahoma, 111 E Chesapeake St., Norman, OK 73019 USA; Point Blue Conservation Science, 3820 Cypress Drive, Suite 11, Petaluma, CA 94954 USA; College of Information and Computer Sciences, University of Massachusetts, Amherst, MA 01003 USA; Department of Computer Science, Mount Holyoke College, South Hadley, MA 01075 USA; Chair in Global Flyway Ecology, Conservation Ecology Group, Groningen Institute for Evolutionary Life Sciences (GELIFES), University of Groningen, PO Box 11103, Groningen, 9700 CC The Netherlands; Department of Ecological Systems Modelling, Institute of Environmental Sciences, Kazan Federal University, 5 Tovarisheskaya, Kazan, 420008 Russia

**Keywords:** Bird migration, FLightR, Hidden Markov models, Particle filter, Solar geolocation, Template fitting

## Abstract

**Background:**

Solar archival tags (henceforth called geolocators) are tracking devices deployed on animals to reconstruct their long-distance movements on the basis of locations inferred *post hoc* with reference to the geographical and seasonal variations in the timing and speeds of sunrise and sunset. The increased use of geolocators has created a need for analytical tools to produce accurate and objective estimates of migration routes that are explicit in their uncertainty about the position estimates.

**Results:**

We developed a hidden Markov chain model for the analysis of geolocator data. This model estimates tracks for animals with complex migratory behaviour by combining: (1) a shading-insensitive, template-fit physical model, (2) an uncorrelated random walk movement model that includes migratory and sedentary behavioural states, and (3) spatially explicit behavioural masks.

The model is implemented in a specially developed open source R package FLightR. We used the particle filter (PF) algorithm to provide relatively fast model posterior computation. We illustrate our modelling approach with analysis of simulated data for stationary tags and of real tracks of both a tree swallow *Tachycineta bicolor* migrating along the east and a golden-crowned sparrow *Zonotrichia atricapilla* migrating along the west coast of North America.

**Conclusions:**

We provide a model that increases accuracy in analyses of noisy data and movements of animals with complicated migration behaviour. It provides posterior distributions for the positions of animals, their behavioural states (*e.g.*, migrating or sedentary), and distance and direction of movement.

Our approach allows biologists to estimate locations of animals with complex migratory behaviour based on raw light data. This model advances the current methods for estimating migration tracks from solar geolocation, and will benefit a fast-growing number of tracking studies with this technology.

## Background

The ability to track animal movements across long distances has revolutionized our understanding of animal ecology and has been helpful to conservation [1, 2]. Until recently, our ability to record this information was limited to larger animals that could carry satellite transmitters. However, recent technological advances have developed miniaturized devices that extend our ability to track much smaller animals, especially migratory songbirds [3]. Solar geolocation data loggers (or geolocators), are simple animal tracking devices that record ambient light levels for the purpose of estimating the latitude and longitude of an animal that wears the device. One of advantage of geolocators is that they typically can record data for a year or longer, *i.e.* cover an annual migration cycle. Despite an ongoing miniaturization of GPS and other satellite-linked tracking devices, geolocators remain useful because their low mass (currently ca. 0.35 g) broadens the range of species that may be tagged [3]. Furthermore, current GPS tracking devices for small birds are limited to a relatively small (*e.g.* 8–10) number of locations that can be recorded, thus it is difficult to use them to generate information on departure and arrival dates. In addition, the simplicity of geolocators design makes them inexpensive, which opens the prospect of affordable population-wide studies of migration and migratory connectivity.

**Fig. 1:**
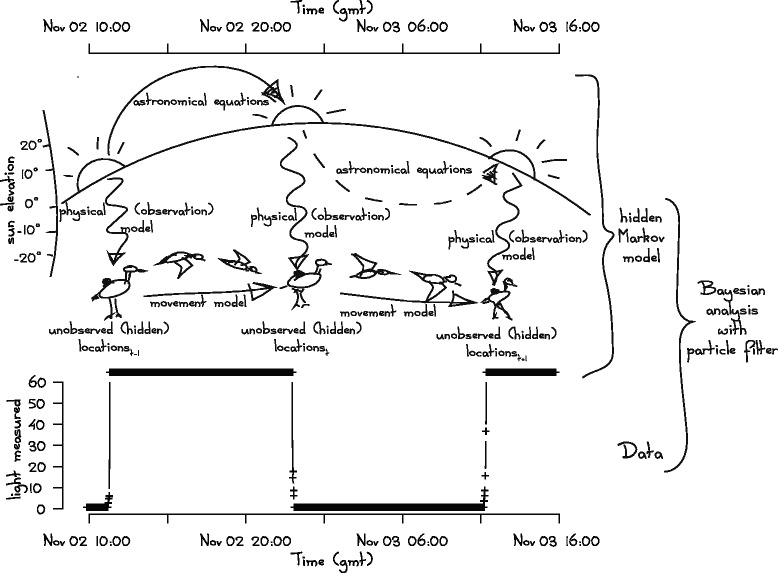
Sketch of solar geolocation principles and our method of analysis. A solar geolocator (shown in black on bird’s back) records light levels and times (example raw *data* in lower panel). When the animal moves, its position is *unobserved (hidden)*, but it can be estimated by the pattern of light changes measured at twilight. We combine a *physical (observation) model* about how light levels change with position and time with some basic knowledge of the patterns of movement between twilights (*movement model*) along with all previous and subsequent positions in a *hidden Markov model* framework. Then, using the *particle filter*, we arrive at the most likely position and movement for each twilight

While the theory of estimating latitude and longitude from the elevation of the sun is not new, the process of reconstructing animal movements by using light intensity levels recorded with geolocators presents several challenges. From the perspective of the hidden Markov modelling framework [4], sketched in Fig. 1, positioning by light level requires at least two parts: (1) an observational model and (2) a process model. In the case of animal positioning we refer to these parts as physical and movement models, respectively. The most common approach to solar geolocation [5] uses a simplified physical model. This model, which is referred to as the ‘threshold method’, requires the definition of each twilight event in a dataset as the time point corresponding to the moment when solar irradiance reaches some arbitrary, but constant, threshold level ([6, 7], see first column in Table 1). Latitude is then estimated by the duration of time between consequent pairs of twilights and the longitude by the time of solar noon or midnight. This threshold approach is still widely used, but it is plagued by many well-known problems such as biased estimates [8], unrealistic assumptions of constant shading, and a null assumption of no movement [9]. Aside from its general simplicity and accessibility, the advantage of the threshold method is that it needs only one data point per twilight period, which makes it well suited to data storage by tiny and simple tags that log a very narrow band of light intensities and have very limited data storage capacity.

**Table 1:** Review of the differences of the existing methods together with rationale for the current contribution

	Customary approach^a^	Ekstrom (2007)	Nielsen & Siebert (2007)	Sumner *et al.* 2009	Current contribution
R package	GeoLight		Trackit	TripEstimation	FLightR^b^
Few points per twilight	+			+−	+
Shading cloud cover free		+		+	+
Optimisation		least squares	UKF	MCMC (block update)	particle filter
Movement allowed			+	+	+
Landscape mask				+	+
Migratory-sedentary switch					+
Behavioural-landscape masks					+
Positions	+ − (not close the equinox)	+	+	+	+
Assesment of precision of estimates			+	+	+
Distribution of possible positions				+	+
Distribution of possible transitions					+

^a^for the details on customary threshold methods one should refer to Hill [44]; Hill & Braun [6]; Ekstrom [7]); ^b^FLightR package (availiable at https://github.com/eldarrak/FLightR) is at the late development stage

Early attempts to improve upon the results of threshold analyses involved the application of a specially developed state-space models on locations estimated with the threshold method [10]. These models served as *post hoc* smoothers and generally improved the estimates, but they did not erase biases because of the original biases in the threshold-based location estimates [11]. In response to the problems of the threshold method stemming from atmospheric properties implicit in the observational model, Ekstrom ([7, 12]; column 2 in Table 1) developed the template fit method. Based on the physics of the atmosphere he derived the relationship between solar angle (angle above the horizon) and near-surface light intensity. Ekstrom [12] showed that the template fit model was robust to the effects of shading (see equations 4–7 in the current contribution). Despite the great potential of the template fit method, it was not used in the next generation of methods nor, to our knowledge, in any published analyses of empirical data. The next generation of methods employed specially developed state space models that not only had a better observational model, but also incorporated assumption of animal movement [13, 14]. These models provide more accurate and precise position estimates and a measure of their uncertainty (see columns 3 and 4 in Table 1).

All the early approaches to solar geolocation were developed for the tracking of marine animals. This specialty called for relatively simple movement models that assumed somewhat constant movement and also incorporated geographic masking under the assumption that marine animals could only occur in the ocean [14] and are not able to move over the land masses. For many terrestrial organisms (*e.g.*, migratory birds) more complex models are needed that can account for prolonged sedentary behaviour interspersed with-long distance movements. Here we describe an approach that attempts to fill the needs associated primarily with tracking small birds by implementing the following features into a state space model: (1) accommodating the narrow band light level data that are recorded by many tags used on birds; (2) allowing for systematic changes in habitats throughout the annual cycle to make the observational model maximally independent from shading regimes; (3) defining behavioural state parameters that describe a bird as either sedentary or migrating; (4) modelling total distance travelled between twilights as opposed to modelling average velocity; (5) masking that allows movements (migratory state) over unsuitable habitats but not settling (sedentary state).

The new analytic framework (implemented in FLightR R package [15]) provides the flexibility to incorporate all of these characteristics. We demonstrate the approach by analysing both simulated and real tracks of small migratory birds.

### Data and models

The example data and a sketch of our approach of inferring tag location are shown at Fig. 1.

### Data

Although the method we outline here is applicable to light level data collected by a wide variety of geolocator tags, we focus our example on data collected by the widely used Mk geolocators developed by British Antarctic Survey (BAS, Cambridge, United Kingdom). Although, BAS has produced several models, they generally save a maximum light level over time intervals ranging from 2 to 10 min (Fig. 1). These tags are optimized for recording low light levels within a narrow band of light intensities around twilight, which results in <10 points collected during each twilight. Thus, a typical dataset will be dominated by minimal values (=0) in response to darkness and maximal values (=64) for measurements in daylight.

To demonstrate our method, we used both real data from BAS tags and simulations of BAS tag data. The real data consist of two tracks. The first is a from a tree swallow *Tachycineta bicolor* tagged at the breeding grounds in southern Canada and flying to wintering grounds in Cuba by the North American East Coast. The second track is from a golden-crowned sparrow *Zonotrichia atricapilla* tagged on its wintering grounds in California and flying to breed to Alaska via the West Coast. The first track features a 2-min and the second employs a ten-minute light logging interval. The simulated data are representative of stationary (not moving) tags, and error associated with weather-related and behavioural shading was derived from real data collected by a tag attached to the above-mentioned tree swallow while it was stationary at a known location.

All existing analytical approaches, including the one presented here, focus primarily on light-level transitions that occur around twilight, so these twilight periods have to be extracted from the data in an automated [13] or semi-automated [5, 16] way. Light-level data collected with geolocators on birds are noisy because birds frequently change their light environment by moving in and out of nest boxes, natural cavities, or dense vegetation, so a completely automated method of detection of twilight events produces many false positives. For this reason we consider a user-controlled, semi-automated process for identifying twilight events in a dataset to be most effective. We used an online interface called TAGS (Totally Awesome Geolocator Service; http://tags.animalmigration.org) to visually inspect data and generate a vector of twilight event periods, both morning and evening, for each day of the year.

### Hidden Markov model

Hidden Markov models are currently the most widely used framework for estimating animal positions and behaviour through time [17, 18]. For solar geolocation the application of hidden Markov models is intuitive as animal movements result in unobserved positions that must be estimated probabilistically (Fig. 1). We developed a model with two parts: the hidden process model of animal movement and the observational model of light measurements from the tag. For easy reference we will refer to these models taken together as the FLightR model.

### Physical observation model

The observational model matches recorded light levels to theoretical expectations at different locations on Earth. During each twilight period *i* the tag records several measurements *j*. Estimation of the theoretical expectation consists of three steps. The first step transforms the coordinates of potential twilight location ***α***_*k*_ at time *τ*_*ij*_ to angle of sun relative to horizon (solar angle, *θ*_*ij*_) with standard astronomical equations [19].

1 $$ {\theta}_{ij}=l\left({\boldsymbol{\alpha}}_k,\ {\tau}_{ij}\right) $$

This step is shared by previous analytical approaches – the Trackit [13] and TripEstimation models [14, 16] and the current contribution.

At the next step, the expected light measurements (*ELM*) are calculated from solar angle. This step varies significantly across analytical approaches, all of which try to account for natural variation in the relationship between solar angle and measured light levels. Sources of this variation are scattering (which includes cloud cover, shading of the tag by landforms and plant leaves; see [9, 20]) and natural variation in refraction [7].

Nielsen and Siebert [13] assumed the function *f* relating solar angle to light measurements was unknown and estimated it using cubic splines with autocorrelation of spline parameters $\varphi \sim$ between consecutive twilights

2 $$ EL{M}_{ij}={f}_{\varphi \sim}\left({\theta}_{ij}\right)+{\varepsilon}_{ij};\kern0.75em {\varepsilon}_{ij}\ \in\ Norm\left(0,\ {\sigma}_i\right) $$

The potential problem of this approach is that errors affecting light intensity are multiplicative, not additive [12]. This means that if light intensity errors are significantly larger than zero the result will be biased. Another drawback is that many light level measurements in each twilight period are required to fit the splines well, and for the widely used BAS geolocators, transitions from the maximum to minimum light-level values usually occur in less than five time steps.

Sumner *et al.* [14] accounted for the multiplicative error by taking the natural log of ELM and fitting the following equation:

3 $$ logEL{M}_{ij}=f\hbox{'}\varphi \sim \left({\theta}_{ij}\right)+{K}_i+{\varepsilon}_{ij};\kern0.75em {\varepsilon}_{ij}\ \in\ Norm\kern0.5em \left(0,\ {\sigma}_i\right) $$

The function *f* ’ is nonlinear and is estimated for each tag with penalized splines on a calibration dataset. The variable *K*_*i*_ is attenuation (or cloudiness) and is also estimated. This approach is less dependent on the shading bias but still requires many light level measurements during each twilight event.

Ekstrom [12] derived the following deterministic relationship (*i.e.* ‘template’) between light at surface (solar irradiance at Earth surface, *SL*) and solar angle *θ*.

4 $$ SL=\left\{\begin{array}{c}\hfill \frac{Cloudiness \times {e}^{-{u}^2}}{1+\mathrm{e}\mathrm{r}\mathrm{f}\left(-u\right)},\kern0.75em u\le 0\hfill \\ {}\hfill \frac{Cloudiness \times {e}^{-{u}^2}}{\mathrm{erfc}(u)},\kern0.75em u>0\hfill \end{array}\right.;\ u=21.5\times \sin \left(\theta \right) $$

where *erf* is the error function and *erfc* is the complementary error function. This equation can be rewritten as follows:

5 $$ \log (SL)= \log (Cloudiness)-\mathrm{u}{\left(\theta \right)}^2- \log \left(\mathrm{erfc}\left(u\left(\theta \right)\right)\right);\kern0.5em u\left(\theta \right)=21.5\times \sin \left(\theta \right) $$

Note that log (*Cloudiness*) plays the same role as the parameter *K* in equation 3.

In the FLightR observational model, we define

$$ {f}^{\hbox{'}\hbox{'}}\left(\theta \right)=-\mathrm{u}{\left(\theta \right)}^2- \log \left(\mathrm{erfc}\left(u\left(\theta \right)\right)\right) $$

Thus

6 $$ \log \left(S{L}_{ij}\right)={f}^{\hbox{'}\hbox{'}}\left({\theta}_{ij}\right)+{K}_i+{\varepsilon}_{ij};\kern0.75em {\varepsilon}_{ij}\ \in\ Norm\left(0,\ {\sigma}_i\right) $$

The function *f ’’* is known, in contrast to the unknown function *f ’* in equation 3.

Equations 4–6 deal with the relationship between the solar angle and light reaching surface of Earth but not to the light measured by a tag. To do that, we need to account for the properties of the tag. Assuming linearity (on a log scale) of the tag measurements we define

7 $$ \log \left( EL{M}_{ij}\right)=I+Z\times \log \left(S{L}_{ij}\right) $$

Where tag intercept *I* and tag slope *Z* are tag specific and do not depend on position or time.

The complete equations for the ELM are then:

8 $$ \log \left( EL{M}_{ij}\right)=Z\times {f}^{\hbox{'}\hbox{'}}\left({\theta}_{ij}\right)+I+K{\hbox{'}}_i + \varepsilon {\hbox{'}}_{ij};\kern0.5em \varepsilon {\hbox{'}}_{ij}\ \in\ Norm\left(0,\ {\sigma}_i\right) $$

Here, the attenuation and noise terms as *K’*_*j*_ and *ε*’_*ij*_ apply directly to log (*ELM*_*ij*_) are not the same as the attenuation and noise terms in eqn. 6. The *observed* light measurement (*OLM*) depends on the attenuation *K’*_*i*_ and slope *Z* that both vary from day to day. The dependency of *K’*_*i*_ on the animal’s behaviour and habitat led us to focus only on *Z* for estimating the likelihood that the observed light measurements were produced at location **α**_k_. We assumed the observations come from a model with tag slope *Z*_*i*_ that is a random variable independent of the animals’ behaviour. This allows us to estimate the distribution of *Z*_*i*_ from the calibration dataset. In all the analysed calibration datasets *Z*_*i*_ had a lognormal distribution:

9 $$ {Z}_i\ \in\ logNorm\left({Z}_{calib},\ \sigma {Z}_{calib}\right) $$

Parameters *Z*_*calib*_ and ${\sigma}_{Z_{calib}}$ are assumed to be constant and are estimated from the calibration at the known *true* sites, where model 8 was fitted.

The complete equations for the observed light measurement are then

10 $$ \log \left(OL{M}_{ij}\right)={Z}_i\times {f}^{\hbox{'}\hbox{'}}\left(\theta ij\right)+I+K{\hbox{'}}_i+\varepsilon {\hbox{'}}_{ij};\kern0.5em {\varepsilon}_{ij}\ \in\ Norm\left(0,\ {\sigma}_{err}\right) $$

In fact, for given location **α**_k_ and twilight *i*, eqn.10 is standard linear model of the form

11 $$ \log \left(OL{M}_{ij}\right)={a}_i+{Z}_i\times {b}_{ij}+{c}_j;\kern0.5em {c}_j\ \in\ Norm\left(0,\ {\sigma}_c\right) $$

with known *b*_*ij*_, unknown *a*_*i*_, unknown *Z*_*i*_, and random error *c*_*j*_ assumed to be normally distributed with a mean of zero. Thus, for any location **α**_k_ we can apply a standard least squares procedure to estimate the mean *Ẑ*_*i*_ and standard deviation ${\sigma}_{Z_i}$ of the unknown *Z*_*i*_ and then use the following probability density as a surrogate for the hypothesis that the data were observed at location **α**_k_:

12 $$ {Z}_i\ \in\ Norm\left({Z}_i\widehat,\ {\sigma}_{Z_i}\right) $$

Thus we can integrate product of density from eqn. 9 and eqn. 12 over all *Z* and estimate the required probability density and the likelihood of data at **α**_k_.

Parameters *Z*_*calib*_ and *σ*_*calib*_ must be estimated for every tag from twilights recorded at a known position. This means that the tag has to be calibrated in a known position for at least 5 days before the animal leaves the area or after it arrives back. The calibration does not need to be a clear-sky calibration and preferably should be done on the bird. The casing around the light sensor may discolour as the tag ages, such that the calibration generated when the tag is deployed will not match the calibration when the tag is recovered. If known locations are available for the beginning and end of the tag use, it is possible to account for ageing by assuming a linear change in the calibrated slope throughout the deployment period.

### Movement model

Previous movement models for solar geolocation were developed for marine animals that are assumed to travel at a relatively constant velocity. For birds, we know that there are long periods (up to 6 months) when birds are primarily sedentary (moving less than several km a day), punctuated by short periods (1–2 weeks) when birds migrate rapidly between breeding and non-breeding locations, but during these periods may spend several days at a single site to refuel before migrating again [21]. For these highly variable movement patterns we needed a more flexible movement model. We used a simplified “Double” model [22, 23] with just two behavioural states: “Sedentary” and “Migrating”. In the context of these two states, we defined the position at the twilight *α*_*i*+1_ as:

13 $$ {\boldsymbol{\alpha}}_0 = \mathrm{initial}\ \left(\mathrm{released}\ \mathrm{location}\right),\ \mathrm{vector}\ \mathrm{of}\kern0.5em \mathrm{coordinates}\left(\mathrm{x};\ \mathrm{y}\right) $$

14 $$ {\boldsymbol{\alpha}}_{i+1}=\left\{\begin{array}{c}{\boldsymbol{\alpha}}_i+{d}_i,\kern0.95em  with\  probability\ {p}_i\\ {}\hfill \kern3em {\alpha}_i,\kern0.95em  with\  probability\ 1-{p}_i\hfill \end{array}\right. $$

Where an offset *d*_*i*_ follows an uncorrelated random walk with a distribution reflecting bird behaviour and described by a set of parameters:

15 $$ {d}_i=\left[\begin{array}{c}\hfill {S}_i\times \cos \left({\Phi}_i\right)\hfill \\ {}\hfill {S}_i\times \sin \left({\Phi}_i\right)\hfill \end{array}\right] $$

At each moment *i*, *d*_*i*_ is distributed as a mixture of a zero increment (no movement) with probability *p*_*i*_, and non-zero increment in direction *Φ*_*i*_ and step length *S*_*i*_. We assigned distributions *Φ*_*i*_ and *S*_*i*_ as follows:

16 $$ {\varPhi}_i \sim \mathrm{vonMises}\left(\varphi,\ k\right) $$

17 $$ {S}_i \sim \mathrm{truncNorm}\left(\upmu,\ \sigma,\ a,\ b\right) $$

For all runs in our models, we assumed that the truncation points were *a* = 45 and *b* = 1000 km. That is, when birds initiated a movement, they could not fly less than 45 km or more than 1500 km in a single between-twilight interval. Parameters φ and *k* in the von Mises distribution reflect direction of migration and its concentration, and the parameters of the truncated normal distribution for distance shape the distribution of inter-twilight flight distances. R packages ‘circular’ [24] and ‘CircStats’ [25] were used to obtain random draws from von Mises distributions given the parameters and ‘truncnorm’ [26] for the truncated normal distribution. All movement was modelled as being uncorrelated in time, assuming that distance flown, direction and behavioural switches during bird migration may be highly independent one day to the next.

Finally, to allow for migration across water bodies, we introduced a spatial behavioural mask that prevents birds from entering a sedentary state in locations corresponding with large water bodies. Hence, birds may fly over water, but cannot switch to sedentary mode in this habitat.

### Bayesian analysis

Many techniques may be used to estimate a hidden Markov model. Our uncorrelated random walk model has five unobserved variables at each of many time steps. Bayesian methods are particularly useful for computing posterior distribution over these variables. The Kalman filter [27] or unscented Kalman filter [28] are other approaches that can be applied to the problem of inference in a long state space model [13], but these do not apply to highly nonlinear posterior distribution patterns caused by non-Gaussian light error distribution with spatially explicit masks. To accommodate these aspects of geolocator data analysis we decided to (1) discretize space and (2) use the particle filter [29] for approximating the posterior distribution. Spatial discretization with creation of a regular grid accelerates computational workflow by minimizing amount of possible between node transitions and also puts no constrains on the spatial error distribution [18, 30, 31]. We used the function regularCoordinates() from the geosphere R package [32] to discretize a state-space into a regular grid with a distance of 50 km between nodes. Choice of the distance of 50 km between grid nodes was arbitrary, and followed the idea that it should be small enough to have posterior probabilities distributed at several grid cells at any time, but at the same time not too small as it will make grid larger and estimation slower. All the movements were estimated between these nodes and the observational model was estimated at these nodes. The approach allows us to “obtain a numerical non-parametric representation of the probability distribution of the animal’s position. This probability distribution illustrates the uncertainty of the estimated movement with a high degree of detail. Finally, we can draw inference about parameters in a likelihood framework, compute the most probable track of the animal, and sample random tracks that the animal may have traveled” [31].

Following Patterson *et al.* [17]*,* we used a particle filter (PF) or ‘sequential importance resampling’. Our implementation of the PF was based on the algorithm from Doucet *et al.* [33] as expanded by Andersen *et al.* [34]*.* The logic of the PF is as follows. At the initialisation phase, our PF creates a sample of 10^6^ particles (points with positions) at the actual release point. Then, for each particle, it generates a new position at *i + 1* from the process model. All new particle locations are then resampled proportionally to their weight (product of the previous likelihoods and the current likelihood, estimated by the observational model and *a priori* constraints as explained below). The resampled set of particles proceeds to the next step and the process repeats. In other words, PF creates 10^6^ possible paths that develop from the release point according to the rules for the movement model. At every twilight all 10^6^ particles are compared to data passed through the observation model. After each check all unlikely particles are replaced by likely ones with the probability of their relative likelihood. Once all iterations are completed, the histories of the remaining particles render a distribution that approximates the spatial probability distribution for the bird at each twilight.

Because of the degeneracy problem common for long PF runs (see *e.g.* [35]), we used block sampling [36] in our PF. assuming that at *n* steps before current step *i* the particles have reached their global optimum. We selected *n* = 90 and then estimated the current weight of the particle as the mean of the logarithms of particle weights at steps [*i-n*]:*n*.

To include information on where the bird was recaptured when the geolocator was removed, we used the approach described by Andersen *et al.* [34]. Here the last *n* states of particle histories were resampled with weights proportional to their probability density function from a normal distribution with a mean of the recapture point coordinates and standard deviation (SD) equal to some measure of precision (because we had 50 km between grid points, we used a SD of 25 km).

*A priori* constraints in the model can be defined according to any biological assumptions. These constraints are used at the resampling stage as weights, and they are not optimised. Here, we present the model with two constraints: a general spatial mask and a behavioural spatial mask. The general spatial mask works through exclusion of selected grid nodes and is binary. Excluding land, for example, may be useful for modelling fish, marine mammal, or pelagic bird movements. The behavioural mask is described above and lowers probability for animals to adopt a stationary state in particular nodes. Value of zero for this mask will completely prevent sedentary mode at the node.

The PF offers a relatively fast optimization technique, but it is unstable to outliers [37]. Outliers in twilight data can happen if an animal stays inside a cavity or nest box during twilight and emerges later, resulting in what would appear to be a late and atypically fast twilight. Ideally such points should be removed during visual inspection of the twilights, but in order to make the PF stable to occasional undetected outliers we have developed two outlier detection approaches. The first approach is used after tag calibration but before the particle filter run. The main idea of the approach is to estimate likely longitude of crossing with the equator for each twilight and then use time series outlier detection software to identify outliers [38, 39]. The other approach is an ‘on the go’ outlier detection technique. This filtering step is implemented at twilight *i* when new particle positions were generated but before the resampling for the next step occurs. At this point, the average distance from *i* − 1 → *i* is compared with *i* → *i* + 1, and if the latter is smaller and mean turning angle at *i* is less the 100°, then twilight *i* is considered to be outlier and its likelihood surface is not used in the particle resampling.

### Application

#### Example 1: simulated stationary tags

We have simulated year-long light data for stationary tags using eqns 9 and 10. The following parameters for the simulations were estimated from real data collected by a tag attached to a Tree Swallow while it had been in a known location for ten days, so they contain a realistic distribution of shading and of slopes.

$$ I+K+Z\kern0.5em \in\ Norm\left(6.14,\ 1.01\right);\ {Z}_{calib} = 0.23;\ {\sigma}_{Z_{calib}}=0.01;\ {\sigma}_{err}=0.32 $$

We began by estimating locations with the threshold method implemented in GeoLight. For this analysis, we used the month of July as the calibration period. Then, using the same calibration period, we estimated the locations in FLightR. We did not use any behaviour or spatial masks and confined our analysis to a reasonably large area with radius of 1000 km around the tag location for the potential spatial extent required for the FLightR runs. For all the simulation and real tag runs we used the same priors of 0.1 for probability of migratory behaviour and 300 ± 150 km for distance covered between twilights. We consider these settings to be suitable for most of the animals except ones that move longer distances between twilights. In our experience with different tags, distance priors do not affect results of the model run, if they are generally correct and wide enough. The probability of migration prior does have an effect, and if selected too high, the resulting track can become noisy. A value of 0.1 was selected as a prior on the basis of good results with simulated data and real tracks; we do not recommend changing it without a specific reason.

**Fig. 2:**
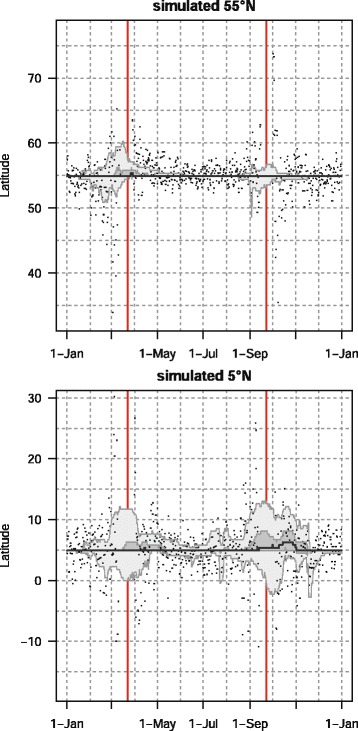
Estimated position of simulated stationary tag by classic approach (GeoLight, points) and FLightR (median estimated latitude with 50 and 95 % credible intervals)

To compare the performances of GeoLight and FLightR, we estimated monthly biases (known minus estimated location) and SD associated with each analysis type for both latitude and longitude (Table 2). Monthly estimates of latitude were not biased when estimated by FLightR (bias ranged from −0.1° to 0.03° due to some rounding error from estimation on a grid) whereas GeoLight biases in latitude estimation ranged from −2.41° to 3.63°. Moreover the FLightR errors on latitude are almost uniform across the year, with SD ranging from 0.23° to 0.33°, whereas the Geolight SD of monthly errors ranged from 1.02° to 10.96° (Fig. 2). Note that the GeoLight method does not provide estimates for latitude at the time around equinox without assumption of stationarity. The results in Table 2 show that estimates from the threshold method are biased while FLightR produces unbiased and consistent estimates.

**Table 2:** Average bias and SD estimated by GeoLight and FLightR for simulated stationary tags at 5° N and 55° N. GeoLight calibration was done by July twilights

Month	5								55							
	Latitude				Longitude				Latitude				Longitude			
	GeoLight		FLightR		GeoLight		FLightR		GeoLight		FLightR		GeoLight		FLightR	
	Mean bias	SD	Mean bias	SD	Mean bias	SD	Mean bias	SD	Mean bias	SD	Mean bias	SD	Mean bias	SD	Mean bias	SD
1	0.13	2.52	−0.03	0.33	−1.05	0.97	0	0.13	0.91	1.63	−0.09	0.26	−0.65	1.74	0.05	0.29
2	0.65	3.57	−0.04	0.28	−1.15	0.83	0	0.13	0.54	3.40	−0.10	0.23	−0.94	1.64	0.04	0.27
3	2.23	10.86	−0.03	0.33	−0.99	0.82	0	0.13	0.85	10.96	−0.09	0.26	−0.93	1.50	0.05	0.29
4	−0.01	6.03	−0.04	0.31	−1.04	0.77	0	0.13	−0.22	2.25	−0.09	0.25	−1.23	1.72	0.04	0.28
5	−0.13	2.46	−0.03	0.33	−0.95	0.83	0	0.13	−0.55	1.68	−0.09	0.27	−1.29	2.47	0.05	0.30
6	−0.64	1.76	−0.04	0.31	−1.15	0.91	0	0.13	−0.90	1.02	−0.09	0.25	−0.63	2.02	0.04	0.28
7	−0.60	2.42	−0.03	0.33	−1.10	0.94	0	0.13	−0.56	1.19	−0.09	0.26	−0.90	1.97	0.05	0.29
8	−0.08	4.74	−0.03	0.33	−1.12	0.99	0	0.13	−0.90	1.62	−0.09	0.26	−1.11	1.43	0.05	0.29
9	3.63	7.88	−0.04	0.31	−1.05	1.02	0	0.13	−2.41	3.59	−0.09	0.25	−0.69	1.46	0.04	0.28
10	0.82	6.45	−0.03	0.33	−1.11	0.67	0	0.13	2.75	8.07	−0.09	0.26	−1.10	1.34	0.05	0.29
11	−0.74	2.82	−0.04	0.31	−0.69	0.86	0	0.13	1.01	2.02	−0.09	0.25	−1.28	1.60	0.04	0.28
12	0.70	2.01	−0.03	0.32	−1.15	0.81	0	0.13	0.76	1.53	−0.09	0.26	−1.15	1.95	0.05	0.29

#### Example 2: tree swallow - 2 min fixes

To demonstrate the application of the FLightR functions to a real-world geolocator dataset, we analysed the track from a single tree swallow that was fitted with an Mk12S geolocation logger on 23 June 2011 at the Long Point Bird Observatory in Ontario, Canada (80.46° W 42.62° N). It was recaptured at the same location and the tag was removed on 7 June 2012. Geolocator was developed by the British Antarctic Survey (Cambridge, United Kingdom) and had 15-mm-long stalk positioned at a 30° angle. The tag recorded maximum light levels at 2 min intervals.

**Fig. 3:**
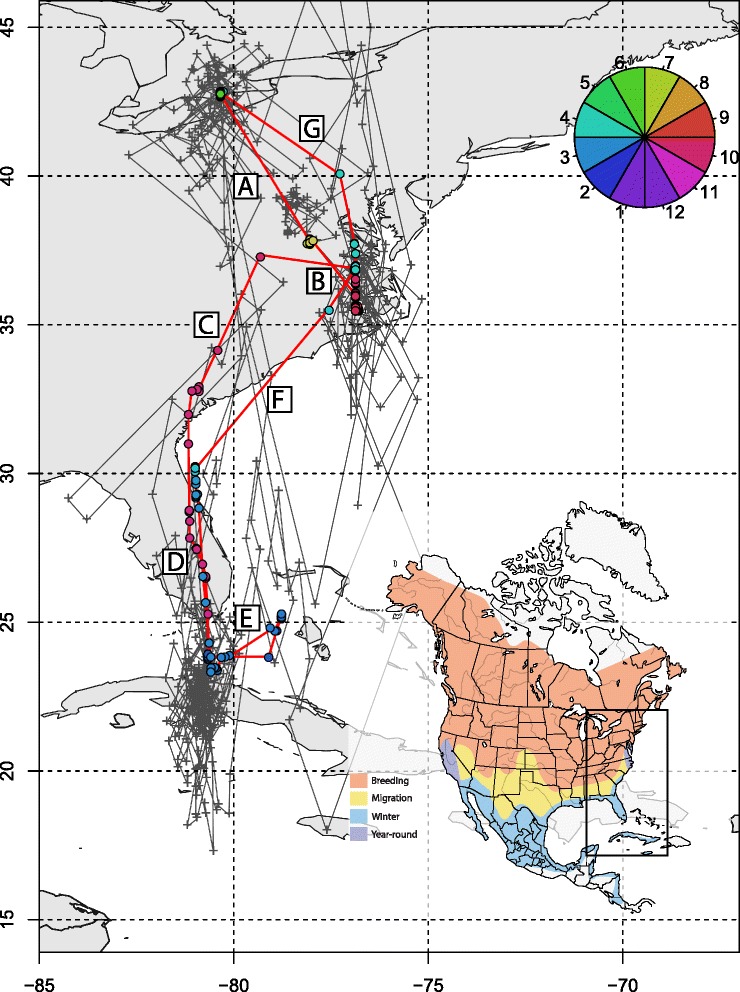
The track of a tree swallow as estimated from 2-min fixing interval data by classic approach (GeoLight, grey line and dots) and FLightR. Inset shows Tree Swallow range in North America. The medians of twilight positions estimated by FLightR are coloured by the month of a year (colours for each month are illustrated with pie chart). In June a bird was tagged on the breeding grounds at the Long Point Bird Observatory in Ontario, Canada. July 19 it left breeding ground and moved to the stopover site in Virginia (segment A). At 1or 2 August it moved to coastal area on the border of North Carolina and Virginia (segment B), where remained stationary until end of October. 23–24 October the bird departed towards a stopover site in South Carolina (segment C), where it stayed for a week and then continued south to Cuba (segment D). The bird remained in Cuba till the end of March, with one exception for flight to the vicinity of the Bimini Islands (segment E). On 28 of March bird left wintering grounds and migrated to the North Carolina/Virginia site (segment F) and after a short stopover there moved straight to the breeding grounds (segment G). Note that no spatial or behavioural masks were used for the FLightR run, so positions were allowed to be everywhere. Raw GeoLight estimates shown on the figure should not be interpreted as a positions and inference can be made only for most likely location during stationary periods, new functions in GeoLight 2.0.1 are available now for the estimation of these most likely stationary locations (Lisovski & Liechti, pers. comm). Tree swallow range image is courtesy of Birds of North America: Cornell Lab of Ornithology

**Fig. 4:**
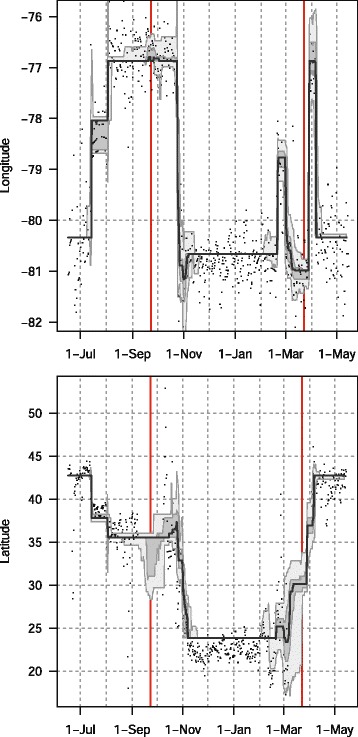
Longitudes (upper panel) and latitudes (lower panel) of a track of a tree swallow as estimated from 2-min fixing interval data by classic approach (GeoLight, dots) and FLightR. The medians of twilight positions estimated by FLightR are shown with accompanying quartile ranges and 95 % credible intervals. Note absence of the latitudinal positions from GeoLight during the equinoxes (shown by red vertical lines)

**Fig. 5:**
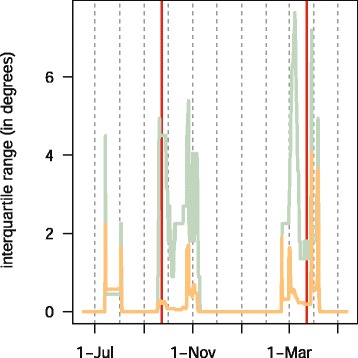
Precision of the estimates of positions by longitude (orange) and latitude (blue) of a track of a tree swallow shown by the interquartile ranges

**Fig. 6:**
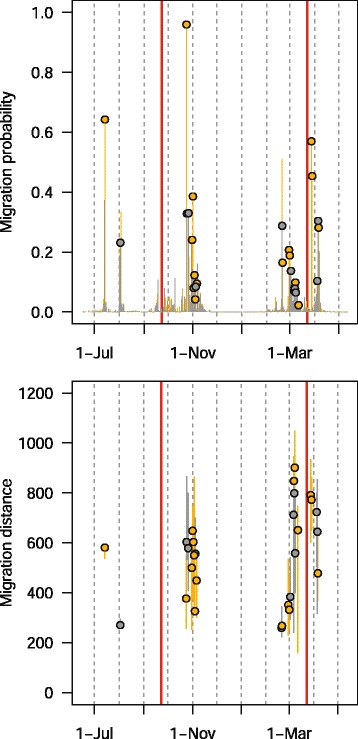
Medians of probability of migration (upper panel) and migration distance with corresponding quartiles (lower panel) for a tree swallow estimated by FLightR. Nocturnal migration is shown in grey and diurnal migration in orange. The circles show transitions which were characterized by shift of the median latitude and/or median longitude. Vertical red lines mark equinoxes

This single track is a small part of a much larger study with many individuals tracked with geolocators at over ten sites across North America (Bradley *et al.* in prep.). We provide raw geolocator data and code to estimate positions for both of the packages online at https://github.com/eldarrak/FLightR/blob/master/examples/tree_swallow_BAS_tag_example/tree_swallow_analysis.Rmd. Note that during the GeoLight run no filters or outlier detection tools were used, and FLightR detected as outliers and excluded data from ~30 twilights. We present four different ways of looking at the path to better reconstruct its details and implications. The reconstructed path from FLightR (coloured symbols in Fig. 3) has the bird staying in the region of Long Point through early July. The bird appears to have stayed at the same longitude and latitude (Fig. 4), and this is backed up with the highest confidence in the reconstructed path (Fig. 5). On the morning of 13 July, the bird departed Long Point (a diurnal departure indicated by the orange dot in Fig. 6a) and made a single flight of over 500 km (Fig. 6b, line segment A in Fig. 3) to a stopover site in Virginia (yellow dots in Fig. 3). It stayed in the same general area until 1 or 2 August, when it departed (segment B Fig. 3) and flew over 200 km to a site near the coastal border of North Carolina and Virginia (Fig. 3). This movement is less certain in its particulars, having interquartile ranges of 1–2 degrees (Fig. 5). The interquartile ranges of position estimates for the following weeks are near the equinox (and thus very unreliable for latitude measures) and vary from low (<1 degree) to very high (>5 degrees; Fig. 5). The bird departed the region on 23 or 24 October, heading south and inland (segment C), and very likely not stopping until reaching coastal South Carolina, where it remained until about 2 November. It then departed for Cuba (segment D), spending most of the rest of the winter there. The one exception to this Cuba winter residency may have been a departure (segment E) to the vicinity of the Bimini Islands on or about 28 February, returning to Cuba about 5 March, but uncertainties at this time of year, nearing the equinox, are very high (Fig. 5). Much more certain is that the winter residency ended on 28 March, when the bird flew from Florida to the coast of North Carolina/Virginia (segment F). It did not stay there for long, if at all, and continued migrating until it reached Long Point on 6 April. The interpretation of the locations during this last northward leg are complicated by the large latitudinal uncertainties associated with the vernal equinox (Fig. 5). The customary approach using thresholds with GeoLight generally agreed with the FLightR estimates.

#### Example 3: golden-crowned sparrow - 10 min fixes

Our sample dataset for golden-crowned sparrow was collected from a bird that was tagged on its wintering grounds in coastal California and tracked to the breeding grounds on the coast of Alaska [40]. The bird was captured and tagged on 2 February 2010 at the Palomarin Field Station in coastal California, United States (37.93° N, 122.74° W). It was recaptured at the same location and the tag was removed on 19 October 2010. The tag was an Mk10S geolocators developed by the British Antarctic Survey (Cambridge, United Kingdom) with a 15-mm-long stalk positioned at a 30° angle. The tag recorded maximum light levels at 10 min intervals. Note that during the GeoLight runs no filters or outliers detection tools were used, and FLightR detected as outliers and excluded about 40 twilights out of 600.

**Fig. 7:**
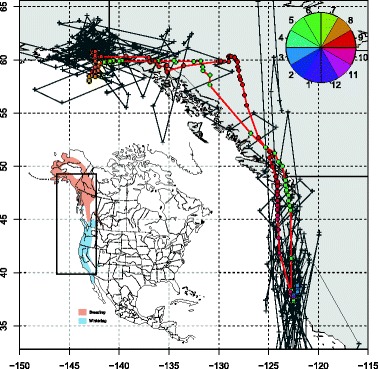
The track of a golden-crowned sparrow as estimated from 10-min fixing interval data by the classic approach (GeoLight, grey line and dots) and by FLightR. Inset shows golden-crowned sparrow range in North America. The medians of twilight positions estimated by FLightR are coloured by the month of a year (colours for each month are illustrated with pie chart). Bird was tagged in 2 February on the wintering grounds in California. It remained close to the capture site till 13 May and then started northward migration. After two weeks of migration it arrived on the breeding grounds (~1 June), and remained there until at least 5 July. It may have moved about 200 km westward after breeding, though the uncertainty here is high. 10 September bird started migration to the wintering grounds, where it arrived at in the end of October. Note that no spatial or behavioural masks were used for the FLightR run, so positions were allowed to be everywhere. Raw GeoLight estimates shown on the figure should not be interpreted as a positions and inference can be made only for most likely location during stationary periods, new functions in GeoLight 2.0.1 are available now for the estimation of these most likely stationary locations (Lisovski & Liechti, pers. comm). Golden-crowned sparrow range image is courtesy of Birds of North America: Cornell Lab of Ornithology

**Fig. 8:**
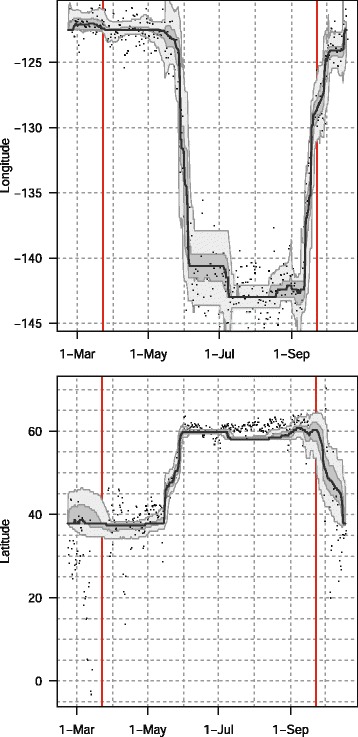
Longitudes (upper panel) and latitudes (lower panel) of a track of a golden-crowned sparrow as estimated from 10-min fixing interval data by classic approach (GeoLight, dots) and FLightR. The medians of twilight positions estimated by FLightR are shown with accompanying quartile ranges and 95 % credible intervals. Note absence of the latitudinal positions from GeoLight during the equinoxes (shown by red vertical lines)

**Fig. 9:**
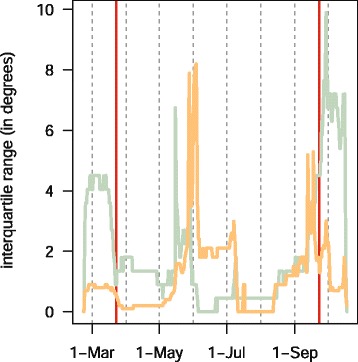
Precision of the estimates of positions by longitude (orange) and latitude (blue) of a track of golden-crowned sparrow shown by the interquartile ranges

**Fig. 10:**
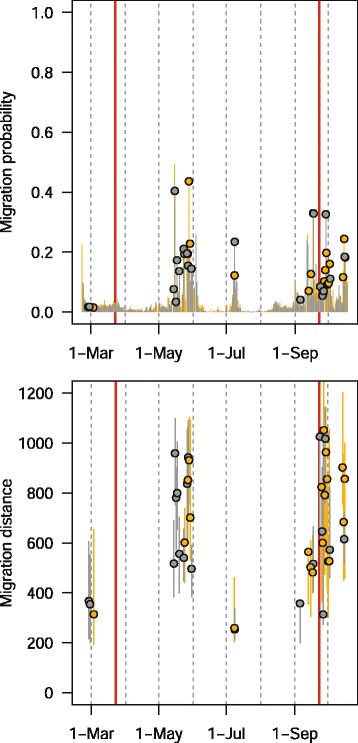
Medians of probability of migration (upper panel) and migration distance with corresponding quartiles (lower panel) for a golden-crowned sparrow) estimated by FLightR. Nocturnal migration is shown in grey and diurnal migration in orange. The circles show transitions which were characterized by shift of the median latitude and/or median longitude. Vertical red lines mark equinoxes

The reconstructed track for the golden-crowned sparrow indicates a movement from the wintering grounds in California, north along the west coast of North America to the breeding grounds in Alaska (Fig. 7). After it was tagged in early February, the bird was sedentary until 18 April, and clear migration activity was initiated May 13 and ceased in early June (Fig. 8). This is consistent with post-tagging observations of the bird that confirmed that it was present (and likely sedentary) at the tagging sight until at least March 31. During the migration both latitude and longitude have high uncertainty (Fig. 9), during the breeding season at Alaska uncertainty in longitude remains high. During spring migration, daily movements were primarily between 500 and 700 km, with some as long as 800 km (Fig. 10). Fall migration occurred between 13 September and 14 October (Fig. 10). During both spring and fall migrations, movements were both diurnal and nocturnal.

Uncertainty in both longitude and latitude increased during both migrations. Notably, uncertainty in longitude also increased around summer solstice in the end of June (Fig. 9). This may be because both twilight lines become almost horizontal and parallel to each other in summer at high latitudes, thus limiting inference on the longitudinal position. The extreme case of this happens at the Arctic Circle (66° N) at solstice. This situation reflects one of the two major limitations in solar geolocation – (1) determination of latitude near equinoxes at any longitude and (2) determination of longitude near solstice at high latitudes. The FLightR model was able to cope with these limitations and provided meaningful positioning results.

## Discussion

### Current model performance

We have presented a new model for the analysis of geolocator data. By analyzing both simulated data and real tracks, we demonstrated that this approach has greater precision than the threshold method (Table 2), which is by far the most commonly applied analysis technique in the literature [41]. The positions obtained for a simulated stationary tag did not have a systematic bias and were reasonably good even near the equinox. For two birds that migrated along the coasts of North America, our approach, without any geographic masks, reconstructed a path that closely matched the coastline. In addition to providing more precise locations, this method provides data on the probability of movement behaviour that can be used to test behavioural hypotheses.

Our method offers several improvements over existing methods for estimating locations from light-level data. To our knowledge, this is the first non-proprietary application that extends template-fitting method all the way from a physical/astronomical model to reconstructing tag locations using timed light-level data. Our method works well on tags that record a relatively narrow band of light intensity data, something that has been problematic with other alternatives to the threshold method [16]. Furthermore, this method applies a movement model that replaces the assumption of constant movement throughout the annual life cycle with a model that allows staying or moving at variable distances at each daily step in the annual path. However, there are still limits to location estimation from light-level data. For actively moving real birds, the estimated latitudes near equinoxes are very imprecise, as there is virtually no latitudinal information in the data at that time. Conclusions about the latitudes of locations during or near the equinoxes must still be made with great caution.

Optimization with current implementation of the particle filter is not fast. One run of the FLightR model takes about 1 h on a four core laptop, which is slower than the Kalman filter approach used in trackit (less than 1 h, [13]) but faster than MCMC in tripEstimation (about 4 h, [16]). The main benefit of the particle filter though is that it not only saves the posterior distribution of particles at each step but also a posterior distribution of all transitions. All of these products are valuable for assessing the confidence and reliability of each of the inferred locations and shifts in behaviour produced by FLightR.

### Drawbacks and directions for further development

The present version of FLightR is very general and could be improved in several ways. The movement part could be improved by introduction of scale-free continuous power-law distance distributions and discrete power-law durations of stopover distributions [42]. The observational model would also be very much improved if we could derive a single general closed-form likelihood equation. The particle filter is not the fastest optimization method, and some other approaches should eventually be tried, such as unscented Kalman filter [28] and the Forward-Backward algorithm [43]. We hope that all these and other potential developments improvements will be facilitated by the current publication and in a few years will bring the field of solar geolocation path reconstruction from its infancy to adulthood.

Another potential area of improvement would be to work with hardware developers to increase the quality of data stored on geolocator tags. Many current tags have variable, and often unmeasured, problems in their lack of calibration, clock drift and change in tag opacity and colour throughout the year. Clock drift is likely to affect the estimated longitudes, while changes in opacity are still not very well understood. Simple calibration procedures and studies of aging effects could help alleviate these problems.

It is perhaps surprising that most geolocators measure only a few points at twilight, which is the most crucial measurement period. The introduction of ‘smart’ recording modes that would allow intense sampling at twilight while skipping uninformative midnight and noon measurements would dramatically improve the precision of location estimation.

### A reflection on the general ‘infancy’ of a burgeoning research field

Solar geolocation publications on migratory birds are booming now in the peer reviewed journals, with rather little attention to the statistical methods necessary to improve the objectivity and precision of path reconstructions. Although some conclusions may be made warranted without statistical approaches (*e.g.* longitude of breeding or wintering grounds), we argue that path-reconstruction of moving animals from noisy light and time data should be done within a statistical framework. Statistical models do not provide exact positions of the bird, and biological interpretations should always be cognizant of estimates of the position and/or timing uncertainty provided by FLightR or any other analysis package. For example, interpretation of the estimated time spent inside a key habitat is much more appropriate than estimating a single fix within that habitat [14].

## Conclusions

Here we introduced a template fitting observational model for solar geolocation as a part of a hidden Markov model. With this approach we estimated positions and migratory schedules of animals accompanied by precision estimates. We believe that the present approach will establish a new benchmark for geolocator analysis that meets the standards applied to most other subfields of research with regard to analytical vigor, objectivity and repeatability.

## Availability of supporting data

Tree Swallow geolocator data, FLightR package and the example of the analysis can be found at GitHub page of the package: https://github.com/eldarrak/FLightR.
